# Hospital and Institutional News

**Published:** 1913-06-28

**Authors:** 


### HOSPITAL AND INSTITUTIONAL NEWS.

### ARE STAFF APPOINTMENTS AT VOLUNTARY HOSPITALS EVER IRREVOCABLE?

We publish in another part of the present issue of The Hospital summaries from a medical and from a legal point of view of the action brought by Drs. Eddowes, Dawson and Knuthsen against St. John's Hospital for Diseases of the Skin to restrain its Board from excluding them from that hospital. The case is to be carried to the Court of Appeal, and pending its decision we refrain from comment.

### THE NEWCASTLE INFIRMARY CHAPEL.

The above has just been reopened, after closure for a very considerable period, to Anglicans only. Our readers will remember the long history of this dispute very well—the expenditure of time and money, the stormy meetings, the legal proceedings. These last indemnified the house committee from the charge of exceeding their powers in providing the chapel for the exclusive use of the Established Church. Apparently encouraged by this, they have now reverted to the *status quo ante*. The expressed wish of the governors of the institution, that the building might serve for the services of Nonconformists as well, presumably remains unchanged. This wish we have before commended as reasonable, and we can only regret the latest turn of affairs. We do not think it at all likely to constitute the final settlement of them, and can only take leave of the subject again for the present with the comment that to spend money on bricks and mortar to provide separately for Dissenters would be extravagant and unjustifiable.

### THE OXFORD CONFERENCE.

By the time that this issue is in the hands of our readers the Annual Conference of the British Hospitals Association, held this year at Oxford, will have begun. Very high praise, and the thanks of all who attend, are due to the Rev. G. B. Cronshaw, who, with the Honorary Secretaries of the Association, has worked extremely hard to make the Conference a success. Much interest is expected from the discussion, which we hope to report next week, on the Insurance Act and its effects upon the hospitals. This, it will be remembered, will follow Sir William Osler's Presidential Address at the first morning session. If the discussion ends, as we have reason for thinking that it will, in the promotion of specific amendments of the Act, this alone will justify the Conference. The previous Conferences have proved abundantly the value of the interchange of ideas and experience which these annual meetings of the Association afford, for, in spite of the efforts which the best institutions make to find out what is being done in the hospital world outside their own localities, there is often still to be found an individual pre-occupation with local affairs which hinders institutional progress. The visits which officers pay from time to time to other institutions are often too brief and too crowded with sight-seeing for the exchange of individual experiences, and one of the chief values of the Conferences of the British Hospitals Association is to give opportunities in the intervals of the sessions for personal conversations and discussions of difficulties which occur at no other time to the same extent.

### NEW SECRETARY TO A MANCHESTER HOSPITAL.

Mr. Herbert J. Dafforne, chief clerk to the house governor and secretary, of Leicester Royal Infirmary, has been appointed secretary to the Ancoats Hospital, Manchester. Mr. Dafforne, who has contributed to The Hospital, has been for three and a half years chief clerk at Leicester, and has been for some eleven years an institutional officer. One of his first hospital appointments was at St. Mary's Hospital, Paddington, where he remained for over four years. Later he was chief clerk to the secretary, Devonshire Hospital, Buxton, for a year and a half before going to Leicester. It need hardly be said that Leicester Infirmary offers one of the best trainings to institutional officers to be found anywhere, and Mr. Dafforne is to be congratulated upon his promotion. He succeeds Mr. Ernest Moffatt.

### BRINGING BERMONDSEY INFIRMARY UP TO DATE.

The Bermondsey Board of Guardians are making efforts to bring their infirmary up to date and to introduce modern methods into their administration. The Medical Superintendent, Dr. E. S. Bell, and his staff have had to surmount many removable difficulties. The purposes of the Guardians to alter and extend the operating-room have received the approval of Sir Arthur Downes, medical officer of the Local Government Board. At present the room is small and the light is bad. A second greatly needed alteration is the transference of the maternity wards from the workhouse in Parish Street to the infirmary. The alteration should be easily carried out, as the Guardians will be able to utilise the ground on which their casual wards were built. It seems a curious mixture—casualty wards adjoining an infirmary and maternity wards in the workhouse—and the sooner that it is changed the better.

### DEATH OF SIR JONATHAN HUTCHINSON.

Sir Jonathan Hutchinson, F.R.S., F.R.C.S., so well known as a surgeon and investigator of leprosy, has died at the age of eighty-four. When he left school he was apprenticed to a York surgeon and at the end of his apprenticeship he became a student at St. Bartholomew's Hospital. After qualifying M.R.C.S., he became house surgeon at York County Hospital. He then returned to London, where he held several staff appointments, intending at the time to become a medical missionary. His marriage in 1856 led him, however, to practise in London, and he became surgeon to the London Hospital. His appointments at four hospitals led to critics describing him as a pluralist, a charge which he rebutted on the ground that he had always advocated additional appointments. In 1862 he published his discovery that the disease then known as strumous ophthalmia was due to inherited syphilis. This disease as a result now became recognised as producing many maladies with which it had not been connected hitherto. In 1869 he visited Norway to study leprosy, in which his acquaintance with individual patients suffering from it had long interested him. He quickly associated it with the use of badly cured fish, a conclusion still combated, though not so whole-heartedly as it once was. South Africa, Ceylon, India, Switzerland, and in 1909 Norway again, were visited in prosecution of these researches. He was also actively interested in the Polyclinic Institution in Chenies Street, often lecturing there, presenting its museum with specimens, and becoming its president. He served on the Royal Commission on Smallpox Hospitals in 1884, and on that upon Vaccination from 1900 to 1906. In 1908 he was knighted, an honour which he had previously declined. He had been President of the Royal College of Surgeons, and of many medical societies. He was an ardent supporter of museums as a factor in education, and built and endowed one at Haslemere, in the neighbourhood of which he became a large proprietor. The popularity of Hindhead as a health resort has been sometimes attributed to him.

### A POINT TO REMEMBER IN CHARITY FETES.

A GARDEN fête, organised for the purpose of clearing off the debt on the Nelson Hospital for Wimbledon and Merton, was held last week in the grounds of Margin House—which is situated on a part of the estate of the late Sir Henry Peek—by kind permission of Mr. Wills. Captain Marryat's father is said to have owned the estate in former years. Behind the house, on one side was the refreshment department; on the other, a large marquee, where, with a variety of articles for sale, were erected stalls. The tea tent was well patronised; but the attractions of the lake in the rear, and the walks surrounding it, probably drew off a good many people who would otherwise have spent a little more money at the stalls. This is a point to remember for future organisers: do not have too attractive surroundings to your stalls. The large lake was illuminated with fairy-lamps after dusk and looked very beautiful. The opening ceremony was performed by Miss Chaplin, daughter of the Right Hon. Henry Chaplin, M.P. for Wimbledon, and the opener was introduced by Mr. Wills, owner of Margin House. The weather was almost perfect, and the two days' fête realised some £650. When expenses are deducted this will go far towards extinguishing the debt which remains on the hospital.

### PANEL LAW IN SCOTLAND.

A VERY curious dispute over the working of the Insurance Act has recently been before a Scottish Court of Law. An insured person, being ill, sent for his panel doctor to attend him. A period of a week elapsed before the doctor came to see him, so meanwhile the insured person called in a private practitioner and sued the panel doctor for £3 11s., of which £2 10s. represented the fees of the doctor who was called in when the paneller could not be obtained. The case for the pursuer was that a contract of service existed between him and the panel practitioner, seeing that he had himself chosen the latter and been duly accepted. He therefore based his claim upon breach of contract. The defence was that no contract existed between the paneller and his individual patients, but only with the local Insurance Committee; and it was urged that the latter alone had power to bring an action for breach of contract. The Sheriff, however, brushed aside this argument, on the ground that the Committee acts on behalf of the insured persons, and that a legal relationship of contract between doctor and patient does definitely exist. That is as far as the action has yet gone, for the pursuer has still to prove the defender's failure to perform the stipulated services, and the damages resulting therefrom. If this decision is adopted also in England, and stands the test of appeal, there is evidently an uncomfortable prospect in view for those who have taken on their panel lists far more insured persons than they can satisfactorily serve.

### COMPLAINTS AGAINST PANEL DOCTORS.

AT a meeting of the London Insurance Committee on Wednesday the Medical Services Sub-Committee prepared a report of the investigations made into the complaints which had been preferred against panel doctors. As to one complaint of neglect against a panel doctor, the Committee express the opinion that he had been drinking when a call at his surgery was made, and that he failed to keep his promises to attend the patient at her address. The Committee conclude that he deserves severe censure, and that he should refund to the insured person her expenses in obtaining treatment from other doctors, and that she should be transferred to another panel doctor. With another doctor who was complained of for not being in attendance at his surgery at the hours he had named, the Committee proposes certain rearrangements, and that no payment be made to him, in accordance with the agreement, until he shall have reimbursed any insured persons for expenses incurred in obtaining treatment elsewhere. If complaints are promptly investigated, and, where justified to the satisfaction of the Committee, promptly brought home to the offenders, the panel practitioners who were rash enough to accept a practically unlimited number of patients may find that there was unwisdom in their greed. It would be a pleasant irony if the authorities who refused to impose a limitation of members were compelled to punish negligences caused by such refusal.

### THE WORK OF THE ORDER OF THE HOSPITAL OF ST. JOHN.

TUESDAY being St. John the Baptist's Day, the members and honorary associates of the Order of the Hospital of St. John of Jerusalem in England held their annual services in the Priory Church of St. John, Clerkenwell, the remains of the choir of the ancient Church of the Order. The Bishop of Southwark, Sub-Prelate of the Order, preached the anniversary sermon. The General Assembly was held in the Chapter Hall at St. John's Gate; Lord Knutsford, Sub-Prior of the Order, presided. Addresses were delivered on the various works carried on by the departments of the Order, notably the Almoners' Department, the British Ophthalmic Hospital at Jerusalem, the St. John Ambulance Association, the St. John Ambulance Brigade, and the Territorial branch, with its voluntary aid detachments. This annual meeting gives the Order an opportunity of reminding the public of its work, which, because varied and many-named, seems never quite appreciated or understood generally. Even hospital men have been known to have the vaguest ideas on the subject.

### SHORTER HOURS FOR ATTENDANTS IN MONMOUTHSHIRE.

In an interesting report on the work of the Monmouthshire Mental Hospital, Dr. J. Glendinning and the committee record a reduction in the hours of duty of the attendants. A petition received from them was presented to a sub-committee, and on its recommendation the following leaves of absence have been granted:—One day a week for three weeks, and on the fourth week one Sunday from 6 A.M. to 10 P.M.; two evenings every week from 6 to 10 P.M.; other evenings from 8 to 10 P.M. when not required for duty. An annual leave of fourteen clear days after two years' service and eight clear days after one year's service has been granted. The effect of this on the members of the staff is the appointment of ten additional attendants, and, including the payment of ration money at the rate of 1s. 1d. per day during the annual holidays to the single attendants, laundry, and kitchen staff, and to the married attendants on their annual leave and on their weekly leave, the total cost is put down at £800 per annum. There is no doubt that the strain of mental work both on men and women workers has been insufficiently grasped, and that we are only at the beginning of reforms in the hours of their work, and in the rates of their pay and superannuation allowances. The concessions granted by the committee of the Monmouthshire Mental Hospital are therefore symptomatic of progress, as well as welcome in themselves.

### THE DIFFICULTIES OF HEAD ATTENDANTS.

"What do you think the most difficult post to fill in a mental hospital?" a medical superintendent was recently asked. "Undoubtedly," he replied, "the post of head attendant. Such a post requires a man who must be on friendly terms with, but yet slightly aloof from, the other attendants, and therefore it does not necessarily belong of right to an ordinary attendant, or form the natural reward of experience and service." How difficult such a post is to the man himself can be illustrated from a somewhat parallel position in the Army. Many Army men refuse promotion from the rank and file to the status of non-commissioned officer because they are afraid of having to behave with official restraint among those who have previously been their equals in rank, and, what is more, actively dislike the idea of increased responsibility. The head attendant in a mental hospital is in the same case. He has to combine the necessary aloofness of authority with a friendliness towards men with whom he may have been living for a long period as his friends and equals. No wonder the medical superintendent has to give anxious thought to filling this post.

### VERANDAHS AT MENTAL HOSPITALS.

A verandah of iron and glass for use in the open-air treatment of women patients suffering from tuberculosis is to be provided at Banstead Mental Hospital, where there are no verandahs at present on the women's side. The cost of providing a suitable structure is estimated at £315, which is, we believe, somewhat above the cost of verandahs at other mental hospitals. The reason is that the only sites furnishing the proper aspect are between two of the large blocks on the first-floor level or over the corridor, and either of these sites will entail additional difficulty in construction. A similar verandah is to be provided at the Claybury Mental Hospital in connection with a ward on the male side for general paralytic cases. It is not intended for those who are bed-ridden, but for those who can walk or are able to be wheeled out in chairs. In this case the cost is estimated at £145. As we said only last week, the verandah system is being adopted more and more for every class of patient.

### THE CHAPLAINCY QUESTION AT SHEFFIELD.

In February last we drew attention to the failure of the Sheffield Guardians to appoint a whole-time chaplain for the Firvale Infirmary. At a meeting of the Guardians last week the Local Government Board administered a rebuke in the form of a letter from which we extract the following: "The Board regret that the Guardians have not seen their way to adopt the suggestion contained in their letter of May 16. The Board adhere to the view that Section 10 of the Poor Law Act, 1879, does not authorise subscriptions as proposed by the Guardians; but if, on further consideration, the Guardians decide to make the payments in question to the Archdeacon of Sheffield and the representatives of the other bodies concerned, the Board themselves will not offer objection." Mr. Newsholme, who in February stated that the visiting chaplain had an impossible task, moved: "That the Board proceed on the lines already laid down, and . . . That the special committee be reappointed to prepare a scheme for the proposed ministrations, and the record suggested be kept and report to the Board thereon." This was carried. In the face of this and the previous discussions why is a whole-time chaplain not appointed, and why has Mr. Newsholme changed his mind?

### SHEFFIELD'S BIG POOR-LAW SCHEME.

The Sheffield Board of Guardians, after an animated discussion, has approved the recommendations of the hospital committee to extend the Firvale Workhouse Hospital. The hospital committee recommended that a children's hospital be built at an estimated cost of £16,819; alteration of the present children's hospital for mental cases at an estimated cost of £1,100; and that a three-storey hospital block be built at an estimated cost of £10,618. Mr. G. T. W. Newsholme, chairman of the Board, explained that the hospital's present accommodation of 643 would be raised by the extensions to 868. Altogether the scheme will cost nearly £40,000, and is supported on two general grounds. The first is that the number of patients in the hospital has continued to rise steadily every year since 1909; and the second is that, while the tendency is to throw more work upon the Poor-Law hospitals, the Poor-Law hospital accommodation in Sheffield is at present inadequate. The scheme is expected to take eighteen months or two years before it will be completed and the new buildings ready for occupation.

### SCHOOL CLINICS OR GRANTS TO HOSPITALS?

Mr. J. A. Pease, M.P., President of the Board of Education, when present at a demonstration on behalf of Rotherham Hospital, Yorkshire, made some remarks about hospitals and the medical inspection of school children which are worth attention. "It is my lot," he said, "to administer grants in connection with the medical inspection and I hope before long to be able to administer grants in connection with the medical treatment of school children." The statistics for the whole country showed, he went on, that a large number of small operations were required in connection with the children attending elementary schools, and these were best undertaken either in clinics specially provided or in the hospitals established in their midst. This meant that they must have separate equipment to attend to all these diseases—a department in connection with the eyes, and others for the throat, skin diseases, ears, and teeth. Then, again, they had preventable diseases which were almost a disgrace to the civilisation in which they lived. Of those who became blind, 40 per cent. were blind through carelessness and negligence at birth. There was also the frightful scourge of consumption raging in their midst. So far the grants given by local authorities to induce hospitals to treat school children have not proved satisfactory. The solution lies in the establishment of school clinics, which, however, must be better supervised than such institutions have been as a rule up to date.

### NEW GENERAL HOSPITAL, TORONTO.

Affiliated to the Provincial University, the new Toronto General Hospital was officially opened last week by Sir John Gibson, Lieutenant-Governor, and Sir James Whitney, the Premier of Ontario. As will be guessed from the preceding sentence, the new General Hospital aims at giving facilities for wide medical teaching in addition to the large accommodation, which makes it, we understand, the largest institution of its kind in the Dominion. Its estimated cost is £800,000, which has been provided in part by two grants of £60,000 respectively from the City and Province. The chairman of the hospital board is Mr. J. W. Flavelle, and, as the *Times* correspondent remarks, he has devoted his best energies and enthusiasm to the great work of starting the new hospital. Although the sums granted by the City and Province are large, it must be remembered that they form together but little more than an eighth of the sum total required. English institutional secretaries may well watch with interest the vitality of voluntary support in Canada.

### THE LIBERTY OF THE MENTALLY DEFECTIVE.

The recent agitation on behalf of the liberty of the subject appears to be having its effect in Committee on the Mental Deficiency Bill. Certificates from two doctors, instead of one, are to be required under Clause 3, which gives power to the parent or guardian of a defective less than twenty-one years of age to place him in an institution. On the question whether there should be an order by a magistrate before the defective could be sent to a home Mr. McKenna said that, recognising that there was great misrepresentation about the matter, he would, before the Report stage, consider words which would give the safeguard asked for, by requiring a judicial order in the case of defectives not covered at present by the Idiots and Imbeciles Act. On the question of presentation of petitions under Clause 5, Mr. McKenna's amendment was accepted, which provided that where a petition was not presented by a parent or guardian the order should not be made without the consent of the parent or guardian unless in the opinion of the judicial authority such consent was unreasonably withheld. How far such modifications will satisfy Mr. J. Wedgwood it is hard to say, for unless the application of the Act is restricted, it seems, he will laugh at all amendment.

### THE HOSPITAL SITE DESCRIBED.

In a new, modern, up-to-date hospital the site is probably the most important factor. In the old days writers possessed the art of description, which is sometimes sadly absent in the present day. A recent number of the *Tablet* describes the site of St. Andrews Hospital, however, as follows:—"No finer site could have been secured. A long, level plateau on the crest of Dollis Hill, surrounded by surviving traces of the ancient Forest of Middlesex, presents an unobstructed view on every side. Gladstone Park lies immediately below to the south; the Weald of Harrow stretches westward; to the east are the heights of Hampstead and Golder's Green; while the Flying Ground of Hendon lies off the northern boundary. Away below Gladstone Park the villages of Neasden and Willesden and the broad vale of Cricklewood lie open to the view; so, for ever secure from all obstruction of light, of air, or of outlook, the site is ideal. The hospital stands on the plateau in the centre of seven acres. Wind-swept, sun-bathed, washed by drifting rains, it is a perfect sanatorium for soul and body." Architects and others in future responsible for site descriptions may possibly take the hint.

### MEMORIAL TO A HOSPITAL SECRETARY.

The establishment of a memorial to the late Mr. Samuel Welsh, who was mainly instrumental in the foundation of the Walsall and District Hospital, and was associated with it as hon. secretary and secretary for nearly fifty years, is being promoted by his daughter, Mrs. Slater, of Hurst Green, Minworth, near Birmingham, who has opened a fund with a subscription of £50. Her aim is to erect in a suitable locality some cottage homes in which nurses with small means who are unable to practise their profession owing to advancing years or failing health may live rent free. This form of memorial was suggested by a more ambitious scheme for the establishment of Homes of Rest for Nurses, in which Mr. Welsh was actively interested prior to his death. A bazaar in aid of the fund is being held at Mrs. Slater's residence this week, and it is hoped that a sufficient sum may be raised from this and other sources to commence the undertaking.

### A MUSEUM OF MEDICAL HISTORY.

On Tuesday last the Historical Medical Museum, which has been organised by Mr. Henry S. Wellcome, was opened by Dr. Norman Moore at 54a Wigmore Street. The Museum, which is to be connected officially with the International Congress of Medicine that meets in August, if Mr. Wellcome's intention is carried out, will form the nucleus of a permanent museum for London. The medical profession, scientists, chemists, and nurses in uniform are alone to be admitted. The scope of the Museum has been outlined previously in these columns, and the exhibits—under Mr. C. J. S. Thompson's arrangement—include objects from the Temple of Maternity in Capua, the illustrated history of our knowledge of tropical diseases, and such curiosities of history as Nelson's medicine chest and Dr. Johnson's spectacles, which three examples give a brief idea of the representative sides of the exhibition. We are particularly glad to note that many portraits and pictures have been included.

### UNIVERSAL SPECIALISTS AND RESEARCH.

Sir Ronald Ross is taking a leaf out of our notebook in complaining that while "there are some half-dozen living Englishmen who have done great work in the investigation of disease, it is characteristic of English administration that the name of not one of these appears on the committees just appointed to administer the first adequate national fund ever laid down in this country for medical research." Only last week we pointed out that the advocates of the scientific study of mental defectives, who recently published a solemn appeal on this head, did not include a single alienist. We endeavoured to explain this by enlarging on the cult of the "universal specialist." Perhaps Sir Ronald Ross can develop the criticism of this cult which we began.

### CLOSING OF BEDS AT WORCESTER INFIRMARY.

The report from the sub-committee appointed to consider the financial position of Worcester General Infirmary has been presented and discussed. The report states that: "After full consideration and after communication with the honorary medical staff, the sub-committee is of opinion, with one dissentient, that the only way by which the expenditure of the infirmary can be brought more into line with the income will be by closing the top floor of the infirmary, and reducing the number of beds in the remaining wards to a maximum of seventy, and recommends the executive committee to carry out the proposal." The Chairman moved the adoption of the recommendation, which was carried. The annual difference between income and expenditure is over £1,000. The opposer of the resolution, Mr. Sidney T. Harris, based his objection on the needs of the sick poor of the city and county—about 1,300 have been treated annually in the infirmary—of whom he calculated with the proposed reduction only 700 could be received; and on the effect of the measure upon the status of the nursing staff. He said that the infirmary could no longer be a training school for nurses. As an alternative, he proposed that efforts should be made to raise the required £2,500 per annum, which he thought should not be impossible in a population of 70,000 or 80,000. Failing that, they might seek by means of an Act of Parliament to obtain leave to sell the £15,000 or £16,000 now ear-marked. He suggested as an amendment that the Hospitals Board be communicated with and asked to call a conference, at which delegates from the infirmary and the City and County Health Insurance Committees should attend to consider how the institution could best be maintained in its present state of efficiency. He found no supporter. The policy of closing beds is a policy of despair, and one which our experience has not taught us to find satisfactory.

### MEDICINE IN A THEOLOGICAL COURSE.

The Rev. Dr. Broughton, who distributed the prizes at the meeting of the London Missionary School of Medicine, which was held at the London Homœopathic Hospital last week, said that, as a doctor of medicine and a priest, if he had to describe the best theological course for a missionary it would include six years of training in some medical university. This valuable testimony, coming from a man whose double qualifications preclude the possibility of bias in his judgment on this point, was illustrated by the remarks of several former students, who explained how much the School's medical training had assisted them in their missionary work. It is difficult to imagine a calling which requires more careful and varied training than that of the missionary, and yet in the past, ignorant zeal has been considered enough, where a knowledge of anthropology and comparative religion, to name but two vast branches of study, are the least that should be demanded. The value of medicine to the missionary is not merely that it gives some chance of his preserving his own life when far away from medical aid, but that it opens the doors to these studies, and shows how many apparent superstitions have a basis which may command the respect, and must the consideration, of anyone wishing to alter them on Western lines. Humility before alien customs rather than any sense of superiority is essential, and that alone a scientific training can give.

### THE LATEST COUNTRY HOSPITAL.

The Children's Home Hospital, formerly at Aberfoyle, built at Strathblane, near Glasgow, was opened last week by the Marchioness of Tullibardine. Country treatment and education are provided for children suffering from tubercular diseases of the bones and joints, who are admitted from the Glasgow infirmaries, the invalid schools, and the Children's Hospital. The institution consists of a single storey, with a verandah along its south front, and comprises in its centre the day rooms, the matron's room, and the kitchen. Two wards, with twelve beds each, on either side of the central block, together with an isolation ward, surgery, and laundry, form the chief factors in the accommodation. The plans have been supervised by Mr. J. Thomson, and are the work of Corvieson and Sons, Glasgow. Lady Tullibardine said that as a result of the institution's work several former patients were earning their living. The educational side of the work was one of its great advantages—an advantage that could not be obtained in a general hospital. The children left without having lost headway in their education. She was glad to know that not only had they treatment in the institution, but that the cases were followed up when the children returned home. There was not only care for the physical health, but also for the development of the mind, and the children were also given an environment of moral and spiritual influences. This would appear to be the latest addition to the country hospitals of the United Kingdom.

### DISPENSERS AND THE INSURANCE ACT.

The result of the official inquiry into the conditions under which medicines are supplied to insured persons is not altogether unsatisfactory from the point of view of dispensers. The inquiry was conducted by a Departmental Committee, which was appointed four or five months ago and has just issued its report. The dispensers who gave evidence before the Committee were Mr. G. Reed, of the Croydon Provident Dispensary, Mr. J. Thompson, a doctor's dispenser, and Mr. F. R. Traynes, dispenser to the Hackney Union Infirmary and hon. secretary of the Association of Certified Dispensers. It was represented to the Committee that, owing to the prohibition in Section 15 of the Insurance Act, whereby arrangements cannot be made for doctors to dispense, part of the work which doctors have employed dispensers to do has been transferred to chemists, and the field of employment open to such dispensers has been restricted in consequence. The Committee, after hearing evidence to this effect, are satisfied that some modification of the present requirements is advisable. They consider that it is possible for persons, who have satisfied a test of training and attainment falling short of that required for registration as a chemist and druggist under the Pharmacy Act, to be employed with safety to dispense in a place of business conducted by a registered pharmacist without acting under his direct supervision. The Committee consider, however, that experience as a dispenser to a doctor or to a public institution should not be regarded as, in itself and alone, qualifying for employment by a chemist and druggist in that capacity. Some direct test of actual attainment should, in their view, be imposed, the chief object of such test being to ascertain the ability of the dispenser to dispense any kind of prescription such as it may afterwards be his duty to deal with. Such a test is, in fact, to be set up by a Bill which, as stated in The Hospital last week, is being drafted by the Pharmaceutical Society. The examination in question should be one which dispensers should not find difficult to negotiate successfully, and there can be little doubt that the position of those who pass it will be considerably better than is the position of doctors' dispensers at the present time.

### A HOMŒOPATHIC DISPENSARY FOR CHILDREN.

A movement is on foot to start a children's homœopathic dispensary at Shepherd's Bush. If it succeeds the dispensary is hoped to grow into a future homœopathic hospital for children, designed to serve the densely populated districts of Hammersmith, Brentford, and Notting Dale. Researches into hospital history show that most of the older institutions sprang from dispensaries in the first place, so, though the custom of starting an institution in this fashion is uncommon nowadays, the present is an instance of its attempted revival.

### THIS WEEK'S DRUG MARKET.

Several changes in the prices of drugs have taken place during the week, but few of them are of much importance. Business continues very quiet, and there seems to be no sign of immediate improvement. The firmer tone noticed in the market for cod-liver oil is maintained, and the general impression is that we shall see higher prices. The present might not be an unfavourable time to make purchases of the oil, in readiness for the consuming season. Coca-leaves have advanced in value, and quotations for cocaine show a higher tendency. Ipecacuanha is rather dearer. There is still no change in the position of quinine, but a definite announcement that the agreement between growers of cinchona-bark and makers of the alkaloid had been signed would not come as a surprise, and would at once have the effect of increasing the value of quinine. Ergot of rye is rather lower in price. Essence of lemon continues to advance in value. Oil of aniseed is dearer, as also is senega-root. Cream of tartar is in good demand. Morphine and codeine have also an easier tendency. Cardamons are dearer. Oil of cloves has again been slightly reduced in price.

